# Enhanced Synaptic-Related Transcriptional Programs in Human Forebrain Organoids for Modeling Synaptopathy-Associated Neurodevelopmental Disorders

**DOI:** 10.3390/cells15201842

**Published:** 2026-10-11

**Authors:** Xiu-Ping Zhang, Ling-Liang Zhang, Xi-Yuan Wang, Shu-Na Wang, Chao-Yu Miao

**Affiliations:** Department of Pharmacology, Second Military Medical University (Naval Medical University), Shanghai 200433, China

**Keywords:** forebrain organoids, cerebral organoids, neural differentiation, brain development, synaptic plasticity, transcriptome

## Abstract

**Highlights:**

**What are the main findings?**

• Human forebrain organoids generated via triple SMAD/Wnt inhibition achieved strict forebrain lineage restriction, faithfully recapitulating human forebrain diverse cell subtypes, transcriptomic signatures, and rudimentary cortical layered structures.
• Human forebrain organoids exhibited enhanced synaptic-related transcriptional programs, while cerebral organoids displayed prominent mid-hindbrain lineage bias and proliferative activity.

**What are the implications of the main findings?**

• The well-characterized forebrain organoid system facilitates standardized organoid construction and offers a promising platform to decipher molecular mechanisms underlying synaptopathies.
• Comparative profiling of forebrain organoids and cerebral organoids provides clear guidance for rational model selection at the transcriptional and morphological levels; forebrain organoids are suitable for investigating forebrain neurons and synaptic-related disorders.

**Abstract:**

Human cerebral organoids (COs) are well-recognized in vitro models for recapitulating human fetal brain development. However, spontaneous unguided differentiation of COs leads to severe regional heterogeneity, limiting standardized research and high-throughput drug screening. In this study, we generated optimized human forebrain organoids (FBs) using a triple SMAD/Wnt inhibition strategy modified from classical CO culture systems. Immunofluorescence staining and bulk RNA sequencing validated the physiological authenticity of FBs and elucidated prominent phenotypic differences between FBs and COs. Molecular analyses confirmed robust and specific forebrain specification in FBs, characterized by sustained expression of canonical forebrain markers and dominant enrichment of neocortical and subpallial signatures, while posterior brain lineage programs were markedly suppressed. Furthermore, FBs faithfully recapitulated diverse fetal forebrain cellular types and rudimentary cortical laminar structures. Transcriptomic profiling revealed that FBs exhibited a marked transcriptional shift toward gene sets associated with neuronal differentiation and synaptic-related biological processes. Gene sets linked to synaptic assembly, transmission, and plasticity were transcriptionally up-regulated, whereas cell-cycle- and proliferation-associated transcriptional programs were downregulated as compared to COs. In conclusion, the optimized FBs faithfully recapitulated key human forebrain developmental features and exhibited enhanced synapse-related transcriptional signatures relative to COs. This refined organoid system provides a valuable in vitro platform for studying forebrain circuit ontogeny and dissecting the molecular pathogenesis of synaptopathy-related neurodevelopmental disorders.

## 1. Introduction

The human forebrain constitutes the primary anatomical substrate for neural circuit assembly and synaptic signaling. Dysregulation of forebrain developmental programs frequently induces synaptic dysfunction, a key pathogenic driver of neurodevelopmental disorders such as autism spectrum disorder and intellectual disability [1,2,3,4]. Direct investigations into human fetal forebrain tissues are hampered by ethical restrictions, while pronounced interspecies differences greatly weaken the translational potential of animal models. Three-dimensional human neural organoids derived from human pluripotent stem cells have become robust in vitro models capable of recapitulating key characteristics of human brain development [5,6]. These models support fundamental research into cortical developmental programs, pathological mechanistic dissection of neurodevelopmental disorders, preclinical therapeutic screening, and regenerative medicine [7,8,9]. The FDA Modernization Act validates human neural organoids as qualified platforms for preclinical evaluation to gradually reduce animal experiments [10,11], making organoids an essential research tool for translational neuroscience.

Classical unguided human cerebral organoids (COs) exhibit heterogeneous patterning, yielding a stochastic mixture of forebrain, midbrain, and hindbrain identities [12,13,14]. Such inter-organoid heterogeneity precludes targeted, precise research focusing exclusively on human forebrain physiology and pathology. Standardized induction protocols for human forebrain organoids (FBs) have not been universally established, and the molecular characteristics distinguishing FBs from COs remain insufficiently elucidated [15,16,17,18]. Furthermore, most current studies adopt COs or roughly induced FBs without systematic phenotypic and molecular validation, which reduces the rationality of model application and hinders their targeted application across disease modeling and mechanistic exploration.

Herein, we successfully generated region-specified FBs using a refined triple inhibition strategy of dual SMAD and Wnt signaling pathways. FBs recapitulated specific forebrain regional identity, diverse cortical cell subtypes, and rudimentary cortical laminar organization. Transcriptomic comparisons between FBs and COs further uncovered a distinctive transcriptional signature within FBs characterized by accelerated neuronal differentiation and elevated synaptic-related transcriptional programs. Specifically, FBs displayed remarkable enrichment in pathways governing synaptic assembly, chemical synaptic transmission, and synaptic plasticity, whereas cell cycle and proliferative gene programs were significantly suppressed. Collectively, this study established a well-characterized human forebrain organoid model with stable regional specification and enhanced synaptic-related transcriptional programs. This optimized model provides a reliable experimental platform to dissect the molecular mechanisms underlying human forebrain corticogenesis and synaptogenesis and holds promising application value for subsequent mechanistic research and translational exploration of synaptopathy-linked neurodevelopmental disorders.

## 2. Materials and Methods

### 2.1. Human Embryonic Stem Cell Culture

Human embryonic stem cells (hESCs, H1 cell line, WA01) were obtained from WiCell (Madison, WI, USA) and cultured following previously established protocols with minor modifications [19,20]. Briefly, six-well plates were pre-coated with Matrigel (Corning, Bedford, MA, USA; Cat# 356234; 1:50 dilution in DMEM, Corning, Cat# 10-013-CV) and stored at 4 °C. Dissociated hESCs were seeded onto Matrigel-coated plates and maintained in mTeSR™1 medium (STEMCELL Technologies, Vancouver, BC, Canada; Cat# 85850) supplemented with 10 μM blebbistatin [21] (Selleck, Houston, TX, USA; Cat# S7099). The medium was refreshed daily with blebbistatin-free mTeSR™1. At 70–80% confluence, cells were dissociated using TrypLE Express (Gibco, Grand Island, NY, USA; Cat# 12605010) and passaged at a 1:6 split ratio in blebbistatin-containing mTeSR™1. All hESC procedures complied with standard guidelines for human embryonic stem cell research.

### 2.2. Human Forebrain Organoids Culture Method

Human forebrain organoids were generated with minor modifications to previously published human COs and forebrain-patterning protocols [12,22]. Our FB protocol builds upon the classical unguided CO method [12], retaining its core culture timeline and commercial medium system while incorporating a triple-inhibition strategy for stable forebrain patterning [22]. Key inhibitor concentrations and treatment schedules were adopted from reported forebrain-patterning protocols. Two main procedural adjustments were implemented: a higher concentration of 50 μM ROCK inhibitor was used to improve single-cell survival during aggregate formation, and commercial N2 supplements replaced manually formulated N2 medium to minimize batch-to-batch variability. A side-by-side comparison of our organoid induction protocol with previously published protocols is provided in Supplementary Table S1.

Dual SMAD inhibition, or single SMAD inhibition combined with Wnt pathway repression, are commonly applied to drive forebrain fate specification in human brain organoids [23]. SMAD inhibition promotes robust neuroectoderm formation, whereas WNT pathway repression dampens posteriorizing signals to favor forebrain identity [23]. Nevertheless, dual SMAD inhibition alone frequently produces appreciable midbrain and hindbrain posterior fates. In contrast, the triple inhibition paradigm supports reliable, robust forebrain fate specification [22]. Accordingly, we adopted the triple inhibition strategy to achieve robust FBs. SB-431542 inhibits the TGF-β/Activin/Lefty signaling pathway, and Noggin represses the BMP signal, synergistically repressing endodermal and mesodermal lineages and promoting neuroectodermal conversion and neural conversion [24]. XAV-939 represses canonical Wnt/β-catenin signaling and has also been implemented in other 3D forebrain organoid protocols to achieve comparable forebrain-specifying effects [22].

Briefly, when hESCs reached 70–80% confluence, hESCs were dissociated into single-cell suspensions using Accutase (Sigma-Aldrich, St. Louis, MO, USA; Cat# A6964) at 37 °C for 3 min, centrifuged at 1200 rpm for 5 min, and resuspended in hESC basal medium containing DMEM/F12 medium (Corning; Cat# 10-092-CV), 20% Knockout serum replacement (KOSR) (Gibco; Cat# 10828028), 1% GlutaMAX supplement (Gibco; Cat# 35050061), 1% MEM non-essential amino acids (MEM-NEAA) (Gibco, Cat# 11140050), 100 μM 2-Mercaptoethanol (Millipore, Burlington, MA, USA; Cat# M3148), and 1% Penicillin-Streptomycin (Gibco; Cat# 15240062). This basal medium was further supplemented with 4 ng/mL recombinant human basic fibroblast growth factor (bFGF, Peprotech, Cranbury, NJ, USA; Cat# 100-18B) and 50 μM ROCK inhibitor Y-27632 (Selleck Chemicals, Houston, TX, USA; Cat# S1049) [12,25]. Subsequently, the prepared single-cell suspension was seeded into ultra-low-attachment 96-well plates (Costar, Carlsbad, CA, USA; Cat# 7007) at a live cell density of 7 × 10^4^ cells/mL, with a final volume of 150 μL medium per well, and incubated in a CO_2_ incubator. On Day 2, spontaneously formed embryoid bodies were treated with forebrain induction inhibitors, achieving final concentrations of 10 μM SB-431542 (Selleck Chemicals; Cat# S1067), 250 ng/mL Noggin (PeproTech, now Gibco/Thermo Fisher Scientific, New Jersey, NJ, USA; Cat# 120-10C), and 3.3 μM XAV939 (Selleck Chemicals; Cat# S1180). On Day 4, full medium replacement was performed with inhibitor-containing medium without bFGF and Y-27632. On Day 6, organoid aggregates were collected, filtered through 40 μm cell strainers, and transferred to ultra-low-attachment 24-well plates in inhibitor-supplemented neuronal induction medium (NIM). Basal NIM was composed of DMEM/F12, 1% N2 (Gibco; Cat# 17502048), 1% GlutaMAX, 1% MEM-NEAA, and 1 μg/mL heparin (Sigma-Aldrich; Cat# H3149). On Day 11, organoids were embedded in 30 μL of Matrigel. After solidification, cerebral organoid differentiation medium (CODM) was added for sustained culture. CODM was composed of 50% DMEM/F12, 50% Neurobasal medium (Gibco; Cat# 21103049), N2, B27 (Invitrogen, Carlsbad, CA, USA; Cat# 12587010), GlutaMAX, MEM-NEAA, 2.8 μg/mL insulin (Sigma-Aldrich; Cat# I9278), and 50 μM 2-Mercaptoethanol, renewed every two days. On Day 15, organoids were transferred to rotating bioreactors in cerebral organoid maintenance medium (COMM). COMM shared identical basal components with CODM, except that the B27 supplement was switched from vitamin A-free B27 (CODM, Invitrogen; Cat# 12587010) to vitamin A-containing B27 (Invitrogen; Cat# 17504044). From Day 45 onward, 1% diluted Matrigel was supplemented into COMM to support long-term maturation.

### 2.3. Human Cerebral Organoids Culture Method

**Figure 1:**
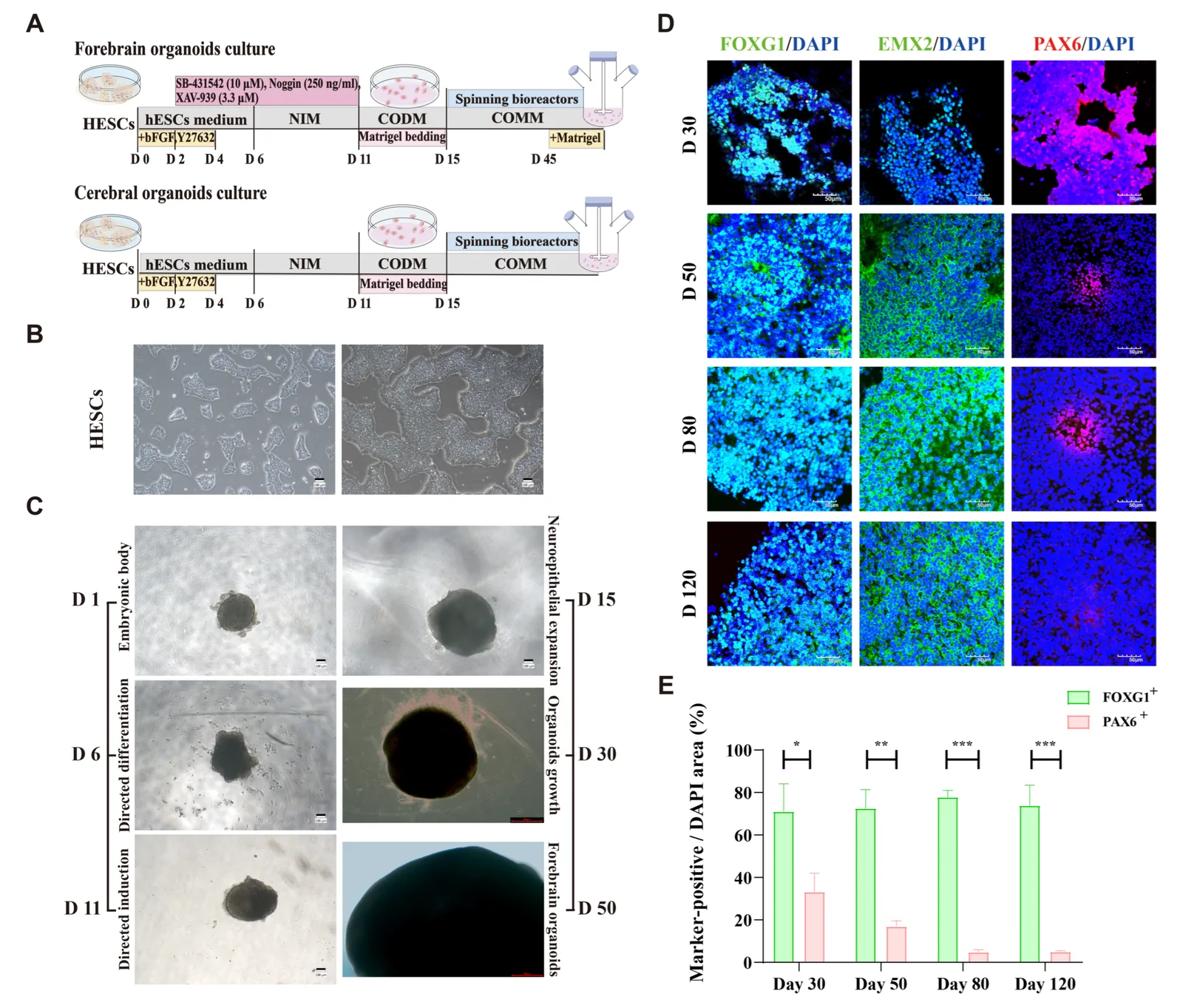
Generation of human forebrain organoids. (**A**) Schematic overview of the culture protocols for human forebrain organoids (FBs) and cerebral organoids (COs). The triple-inhibitors consisting of SB-431542, Noggin, and XAV939 were supplemented exclusively in the FBs culture system from Day 2 to Day 11 for directed forebrain patterning. No inhibitors were added in the CO protocol. HESCs, human embryonic stem cells; NIM, neuronal induction medium; CODM, cerebral organoid differentiation medium; COMM, cerebral organoid maintenance medium. (**B**) Representative bright-field micrographs showing the typical morphology of undifferentiated hESCs prior to organoid induction. Scale bars: 100 μm. (**C**) Morphological characteristics of FBs across multiple developmental stages, from embryoid body formation (Day 1) to forebrain organoid differentiation (Day 50). Scale bars: 100 μm for Day 1, Day 6, Day 11, Day 15; 500 μm for Day 30 and Day 50. (**D**) Representative immunofluorescence staining of canonical forebrain-specific markers (FOXG1, EMX2, and PAX6) in FBs at Days 30, 50, 80, and 120. Representative immunofluorescence images derived from two independent differentiation experiments. A total of 3 organoids were assessed per time point. Scale bars: 50 μm. (**E**) Quantitative analyses of FOXG1^+^- and PAX6^+^-positive area in FBs at Days 30, 50, 80, and 120. Data represent mean ± SEM. Two-way ANOVA followed by Tukey’s post hoc multiple-comparison test. Asterisks indicate significant differences between FOXG1^+^- and PAX6^+^-positive area at the same time point. * Adjusted *p* < 0.05, ** Adjusted *p* < 0.01, *** Adjusted *p* < 0.001. Quantitative analysis was performed using *n* = 3 independent organoids per time point, obtained from two independent differentiation experiments.

Classical COs were generated as previously described [12]. The key difference was the complete absence of SMAD and Wnt pathway inhibitors throughout differentiation (Figure 1A). Briefly, hESCs formed three-dimensional aggregates spontaneously without directed patterning treatment. Medium replacement, organoid aggregate collection, Matrigel embedding, and long-term bioreactor culture were performed identically to the FB protocol.

### 2.4. Cryosectioning and Immunofluorescence Staining

Organoids were washed with PBS, fixed in 4% paraformaldehyde (Biosharp, Suzhou, China; Cat# BL539A), and dehydrated in 30% sucrose solution at 4 °C. Samples were embedded in freezing medium (Leica, Heidelberg, Germany; Cat# 14020108926) and cut into serial sections at 10 μm thickness using a freezing microtome (Leica, Heidelberg, Germany; CM3050S). Sections were stored at −80 °C until use.

For immunofluorescence staining, cryosections were baked, permeabilized with 0.25% Triton X-100, and blocked with 10% goat serum (Beyotime, Shanghai, China; Cat# C0265) for 2 h. Subsequently, sections were incubated overnight with primary antibodies at 4 °C against FOXG1, PAX6, SOX2, HOPX, PTPRZ1, TBR2, CTIP2, and SATB2. After PBS washing, sections were incubated with Alexa Fluor 488/594 secondary antibodies for 90 min at room temperature. Full details for all antibodies are provided in Supplementary Table S2. Nuclei were counterstained with DAPI (Beyotime, Cat# C1006). Finally, sections were mounted with anti-fade medium (Beyotime, Cat# C0126) and imaged using an FV1000 confocal microscope (Olympus, Tokyo, Japan).

### 2.5. Bulk RNA Sequencing and Data Analysis

Forebrain organoids were harvested at Days 30, 50, and 80, while COs were harvested at Day 50, and lysed in TRIzol (Invitrogen; Cat# 15596026CN). Library construction and bulk RNA sequencing were performed commercially. All downstream bioinformatic analyses were performed using R software (version 4.5.2).

Total RNA was extracted using the Magzol RNA Isolation Kit (Magen, Guangzhou, China; Cat# R4801), treated with RNase-Free DNase Set (QIAGEN, Hilden, Germany; Cat# 79254). RNA concentration was quantified by Qubit (Thermo Fisher Scientific), and purity was determined by NanoDrop ND-2000 (Thermo Fisher Scientific), with an acceptable A260/A280 ratio ranging from 1.8 to 2.1. RNA integrity was assessed using the Agilent 2100 Bioanalyzer/Agilent 4200 TapeStation (Agilent Technologies, Santa Clara, CA, USA); only samples with RIN ≥ 6 were used for library preparation. RNA-seq libraries were prepared from qualified RNA using the VAHTS Universal V6 RNA-seq Library Prep Kit for Illumina (Vazyme, Nanjing, China; Cat# NR604-02). Paired-end 150 bp sequencing was conducted on the NovaSeq 6000 platform (Illumina, San Diego, CA, USA). Clean reads were mapped to GRCh38 using Hisat2 (v2.0.4), and gene expression was quantified with Stringtie (v1.3.0) to generate gene count matrices.

Batch-effect correction was performed using ComBat-seq, and adjusted counts were solely used for visualizing the canonical forebrain cell-type-associated transcription factors collected at Days 30, 50, and 80. For this longitudinal dataset, a batch was defined as an independent NovaSeq 6000 sequencing run, corresponding to three separate sequencing runs for different time-point samples. No batch correction was performed for the Day 50 FBs versus COs comparison, as these samples were derived from a single differentiation experiment and sequenced within the same sequencing run. Full sample metadata, including sample identifiers, grouping information, sampling time points, and differentiation batch and sequencing-run labels, are provided in Supplementary Table S3.

Differentially expressed genes (DEGs) between FBs and COs at Day 50 were identified using limma (v3.65.3) with voom transformation and empirical Bayes moderation. After deduplication of duplicated gene names, 50,464 genes were retained. Genes with zero counts across all samples were discarded, resulting in 31,249 genes. We further retained genes with non-zero counts in at least 4 out of 6 biological samples, leaving 23,986 genes. Subsequent low-abundance filtering was performed on TMM-normalized CPM values; only genes with CPM > 1 in at least three samples were retained for downstream analysis. Finally, 16,965 genes were included for differential-expression testing. The mean-variance dispersion relationship derived from voom transformation was examined to assess the adequacy of voom weight estimation (Supplementary Figure S1). Genes were defined as differentially expressed with |log_2_FC| ≥ 1 and adjusted *p* < 0.05. Principal component analysis (PCA) was performed and unsupervised clustering heatmaps were generated to illustrate global transcriptional differences. Volcano plots were constructed to visualize the distribution of DEGs, in which the top 20 significantly upregulated genes associated with upper-layer neurons and GABAergic interneurons were annotated using ggrepel (v0.9.6). Canonical forebrain cell-type marker profiles were visualized to compare cellular composition between groups using ggplot2 and pheatmap. The marker set combined manually curated canonical cell-type signatures and prefiltered high-confidence DEGs, as illustrated in Supplementary Table S4. GO and KEGG enrichment analyses were performed on upregulated and downregulated DEGs using clusterProfiler (v4.18.0), with FDR < 0.05 as the significance threshold. The top 10 enriched GO terms and all significant KEGG pathways were exhibited as bubble plots and bar plots, respectively. Hallmark gene set enrichment analysis (GSEA) was performed using pre-ranked transcriptome profiles with Benjamini–Hochberg correction.

For regional signature evaluation, human fetal brain region-specific gene sets (12–24 post-conception weeks [pcw]) were obtained from previously published datasets, and subpallium-related gene sets were additionally supplemented from the Allen Human Brain Atlas (Supplementary Table S5) [22,26]. Regional GSEA was performed using fgsea with 5000 permutations, and representative region-specific genes were visualized by heatmaps.

A curated synaptic gene set was constructed by integrating GO and SynGO database annotations, covering synapse assembly, synaptic transmission, LTP, synaptic plasticity, and neural circuit development (Supplementary Table S6) [27]. After deduplication, unified synaptic gene collections were used for targeted GSEA with 1000 permutations. Significant synaptic pathway enrichment was visualized by bubble plots and running score curves. DEGs (FDR < 0.05, |log_2_FC| ≥ 1) within core synaptic programs were extracted, Z-score normalized, and displayed in heatmaps to illustrate synaptic transcriptional differences between FBs and COs.

### 2.6. Statistical Analysis

Statistical analyses were performed using GraphPad Prism 9 (GraphPad Software, San Diego, CA, USA). All quantitative data were presented as means ± SEMs. Statistical analyses were conducted using two-tailed Student’s *t*-test or two-way ANOVA followed by Tukey’s post hoc test for multi-group comparisons. A *p*-value < 0.05 was considered statistically significant.

## 3. Results

### 3.1. Generation and Stable Regional Specification of Human Forebrain Organoids

Diverse combinatorial regimens of transforming growth factor-β (TGF-β) and Wnt signaling inhibitors administered at defined developmental time windows have been widely used to drive forebrain lineage commitment [28,29]. TGF-β inhibitors, the canonical and well-recognized SMAD antagonists, encompass BMP and Activin/Nodal signaling inhibitors [23]. Nevertheless, conventional protocols relying on single or combined SMAD and Wnt inhibition often generate FBs with unstable regional identities and poorly defined molecular profiles, and a standardized protocol for generating highly forebrain-specified FBs remains lacking.

Here, we constructed optimized FBs by adopting a modified triple inhibitor induction strategy consisting of SB-431542, Noggin, and XAV939, adapted from classical COs culture procedures (Figure 1A). The three inhibitors simultaneously suppress SMAD and Wnt signaling during ectodermal patterning and early neural specification to drive and stabilize forebrain lineage specification. Briefly, hESCs at 70–80% confluence were dissociated and seeded into ultra-low-attachment plates to form cell aggregates (Figure 1B). The three inhibitors were supplemented from Day 2 to Day 11 to guide ectodermal differentiation and forebrain-oriented patterning. These aggregates were embedded in Matrigel and cultured in inhibitor-free CODM on Day 11, followed by rotary suspension culture on Day 15 to facilitate organoid maturation. This stepwise guided forebrain differentiation system supported steady organoid growth, and the derived FBs consistently exhibited a uniform, compact spherical morphology throughout the entire culture period (Figure 1C). Immunofluorescence staining confirmed sustained expression of canonical forebrain markers (FOXG1, EMX2, PAX6) in FBs across multiple developmental stages (Days 30, 50, 80 and 120), confirming the high stability and reliability of forebrain regional specification (Figure 1D). We quantified the proportions of FOXG1^+^- and PAX6^+^-positive area in FBs at Days 30, 50, 80 and 120 of differentiation (Figure 1E). Two-way ANOVA was applied with differentiation time and cell marker as two factors. The main effect of the marker was significant (*F*_(1,16)_ = 125.4, *p* < 0.001), demonstrating that the proportion of FOXG1^+^-positive area was overall markedly higher than that for the PAX6^+^-positive area. Neither the main effect of the differentiation time (*F*_(3,16)_ = 1.122, *p* = 0.3696) nor the time-by-marker interaction reached statistical significance (*F*_(3,16)_ = 2.263, *p* = 0.1204). Thus, the overall proportion of marker-positive area did not change significantly across the examined time window, and the temporal patterns of FOXG1^+^ and PAX6^+^ signals were statistically indistinguishable. Quantitative measurements revealed that FOXG1^+^-positive area remained persistently high throughout differentiation, whereas the PAX6^+^-positive area showed a gradual descending trend from Day 30 to Day 120. Tukey’s multiple-comparison post hoc test further confirmed significant differences between FOXG1^+^ and PAX6^+^ within identical differentiation time points.

### 3.2. Physiological Authenticity of Human Forebrain Organoids

**Figure 2:**
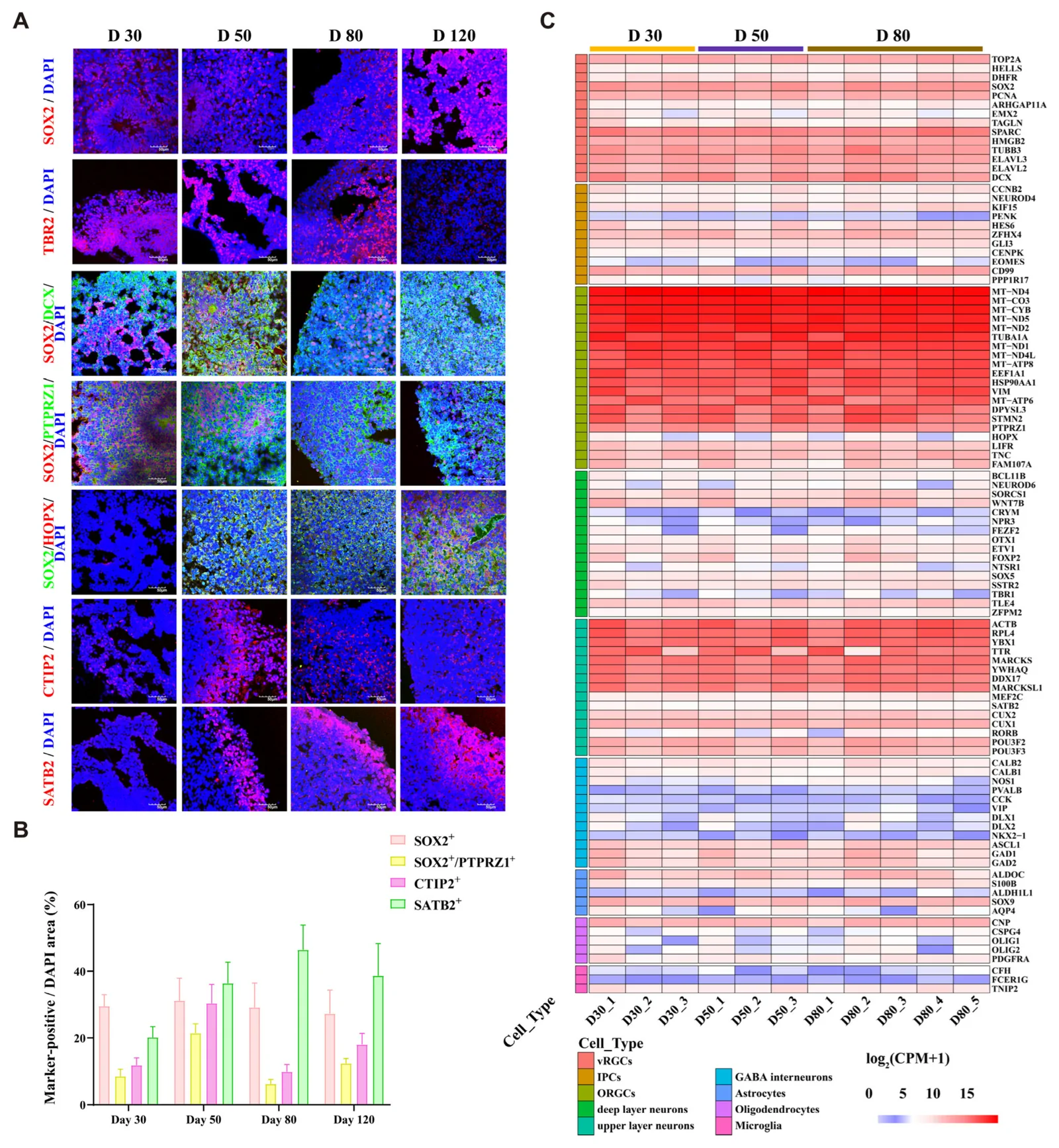
Human forebrain organoids recapitulate diverse cortical cell subtypes. (**A**) Representative immunofluorescence staining of cortical cell subtype markers in forebrain organoids at Days 30, 50, 80, and 120. Markers include SOX2 (neural stem cells), TBR2 (intermediate progenitor cells, IPCs), SOX2/PTPRZ1 (outer radial glial cells, oRGCs), DCX (immature neurons), CTIP2 (deep-layer neurons), and SATB2 (upper-layer neurons). Representative immunofluorescence images were derived from two independent differentiation experiments. A total of 3–5 organoids were assessed per time point. Scale bars: 50 μm. (**B**) Quantitative analysis of SOX2^+^-, SOX2^+^/PTPRZ1^+^-, CTIP2^+^-, and SATB2^+^-positive area in FBs at Days 30, 50, 80, and 120. Bar plots show the percentage of marker-positive area relative to the total DAPI nuclear area (%) across four developmental time points. Two-way ANOVA was followed by Tukey’s post hoc test. Quantitative analysis was performed using *n* = 3–5 organoids per time point, obtained from two independent differentiation experiments. Quantitative data are presented as means ± SEMs. (**C**) Transcriptomic heatmap showing signature genes of diverse endogenous forebrain cell subtypes, including ventricular radial glial cells (vRGCs), oRGCs, IPCs, deep-layer neurons, upper-layer neurons, GABAergic interneurons, astrocytes, and oligodendrocytes. Gene expression values were normalized to log_2_(CPM + 1). Rows represent cell-type-associated marker genes grouped by cell type, and columns represent individual samples collected at Days 30, 50, and 80. The color scale gradient indicates relative gene expression, with blue indicating low expression and red indicating high expression. All samples (*n* = 3 per time point) were independent organoids derived from a single independent experiment.

To further validate the physiological authenticity of FBs, we characterized the cellular diversity and primitive tissue architecture using immunofluorescence staining and bulk RNA sequencing. As FBs underwent differentiation, diverse cortical cell types were sequentially generated (Figure 2). At Day 30, FBs harbored abundant SOX2^+^ ventricular radial glial cells (vRGCs), TBR2^+^ intermediate progenitor cells (IPCs), SOX2^+^/PTPRZ1^+^ outer radial glial cells (oRGCs), and numerous DCX^+^ immature neurons, whereas SOX2^+^/HOPX^+^ oRGCs were barely detectable at this stage. In addition, neuronal markers, including the deep-layer neuron marker CTIP2 and upper-layer neuron marker SATB2, were detected in limited cell populations within Day 30 FBs, consistent with an early stage of neuronal differentiation. As differentiation proceeded, FBs recapitulated a full repertoire of cortical lineage markers at Day 50, including SOX2 for vRGCs, TBR2 for IPCs, SOX2/PTPRZ1 and SOX2/HOPX for oRGCs, DCX for immature neurons, CTIP2 for deep-layer neurons, as well as SATB2 for upper-layer neurons. These cortical cell populations were stably maintained through Day 80 and Day 120 (Figure 2A). Two-way ANOVA was performed with differentiation time and marker identity as two factors for the quantification of SOX2^+^-, SOX2^+^/PTPRZ1^+^-, CTIP2^+^-, and SATB2^+^-positive area (Figure 2B). The main effect of the marker was significant (*F*_(3,40)_ = 13.85, *p* < 0.001), indicating an overall difference in positive area fractions among these four markers regardless of differentiation stage. The main effect of differentiation time also reached significance (*F*_(3,40)_ = 3.641, *p* = 0.0206), revealing global temporal changes in marker signals across organoid maturation. No significant time-by-marker interaction was found (*F*_(9,40)_ = 1.572, *p* = 0.1571), suggesting that the temporal changing patterns did not statistically differ between markers. Quantitative measurements revealed that SATB2^+^-positive area showed a trend to progressively increase over differentiation, accompanied by relatively stable CTIP2^+^ signal levels. SOX2^+^-positive area remained abundant throughout the culture period, and SOX2^+^/PTPRZ1^+^ double-positive area persisted at relatively low levels at all time points. A heatmap of cell-type-specific transcripts further revealed that FBs expressed gene sets for diverse neural progenitors and neuronal subtypes, reminiscent of molecular profiles of the human fetal forebrain (Figure 2C).

**Figure 3:**
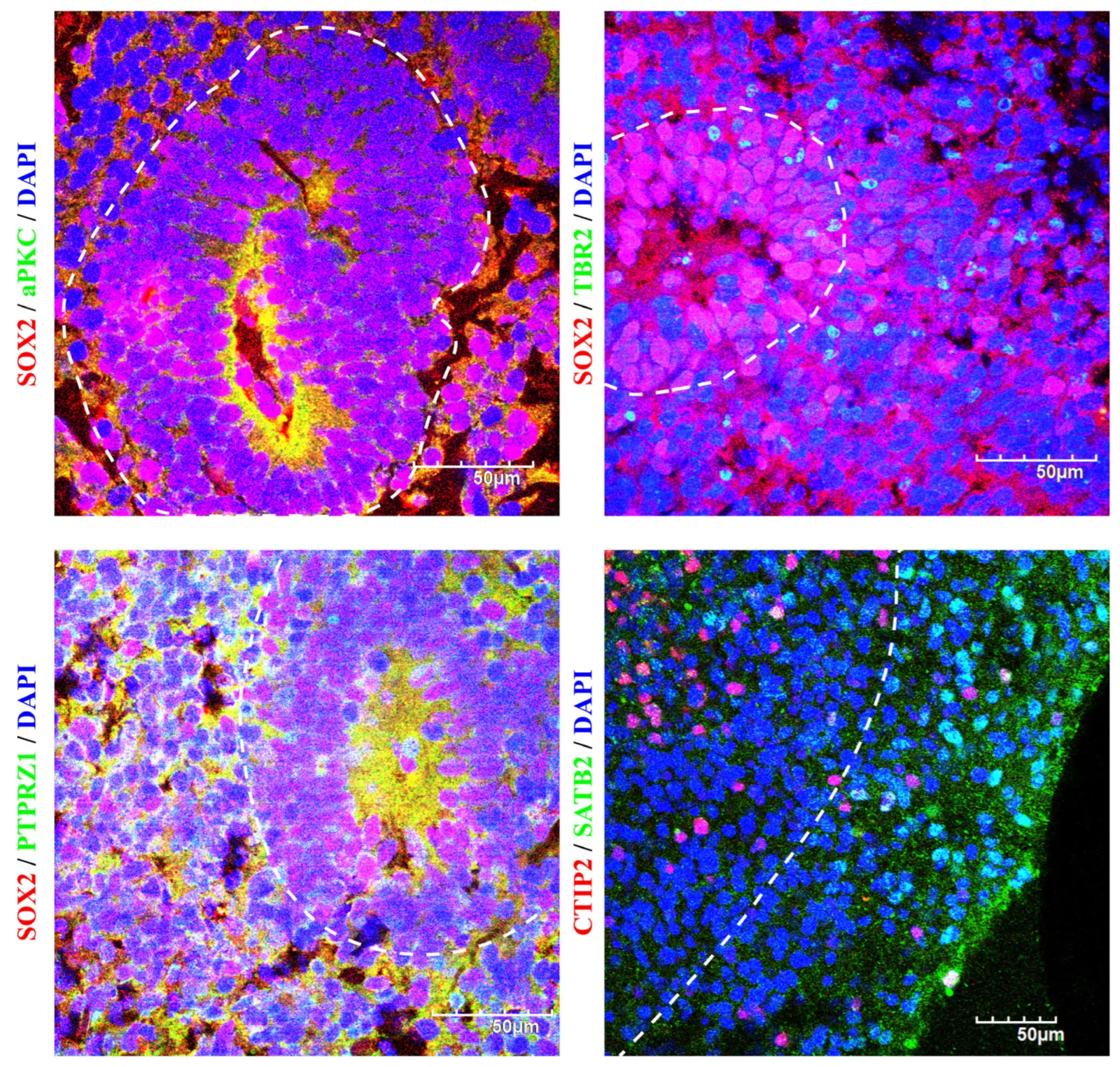
Representative immunofluorescence staining of primitive cortical laminar structures in Forebrain organoids. Organoids exhibited ventricular zone (VZ)-like structures enriched with SOX2^+^ progenitors, with aPKC staining marking the apical luminal surface of neuroepithelial progenitors. Surrounding these domains, the surrounding subventricular zone (SVZ)-like compartment was constituted by TBR2^+^ IPCs and SOX2^+^/PTPRZ1^+^ oRGCs. White dashed lines in the panels separate these inner VZ-like domains from the outer SVZ-like compartments. The outer cortical plate (CP)-like region harbored CTIP2^+^ deep-layer neurons and SATB2^+^ upper-layer neurons. SATB2^+^ neurons occupied a more superficial position compared with CTIP2^+^ neurons, a spatial pattern delineated by white dashed lines, recapitulating the inside-out lamination characteristic of the human fetal cortex. *n* = 3–5 independent organoids per time point, obtained from two independent differentiation experiments, multiple sections imaged per organoid, with randomly selected fields of view. Scale bars: 50 μm.

Notably, the optimized FBs assembled a rudimentary laminar structure resembling the human fetal cortex (Figure 3). Typical neural rosettes densely populated by SOX2^+^ progenitors were widely distributed within organoids, mimicking the embryonic ventricular zone (VZ). Apical-polarity marker aPKC was enriched at the luminal apical surface inside the rosette structure, revealing a well-organized neuroepithelial architecture. TBR2^+^ IPCs and SOX2^+^/PTPRZ1^+^ oRGCs collectively constituted the subventricular zone (SVZ) surrounding the VZ. At the organoid periphery, CTIP2^+^ deep-layer neurons and SATB2^+^ upper-layer neurons assembled well-organized cortical plate-like structures exhibiting canonical inside-out lamination: CTIP2^+^ neurons resided in deeper regions, whereas SATB2^+^ neurons occupied the superficial layers. These results demonstrate that our optimized protocol efficiently generated diversified forebrain cell lineages and faithfully recapitulated the fundamental tissue architecture of the human fetal forebrain.

### 3.3. Phenotypic and Lineage Specification Disparities Between Human Forebrain Organoids and Cerebral Organoids

**Figure 4:**
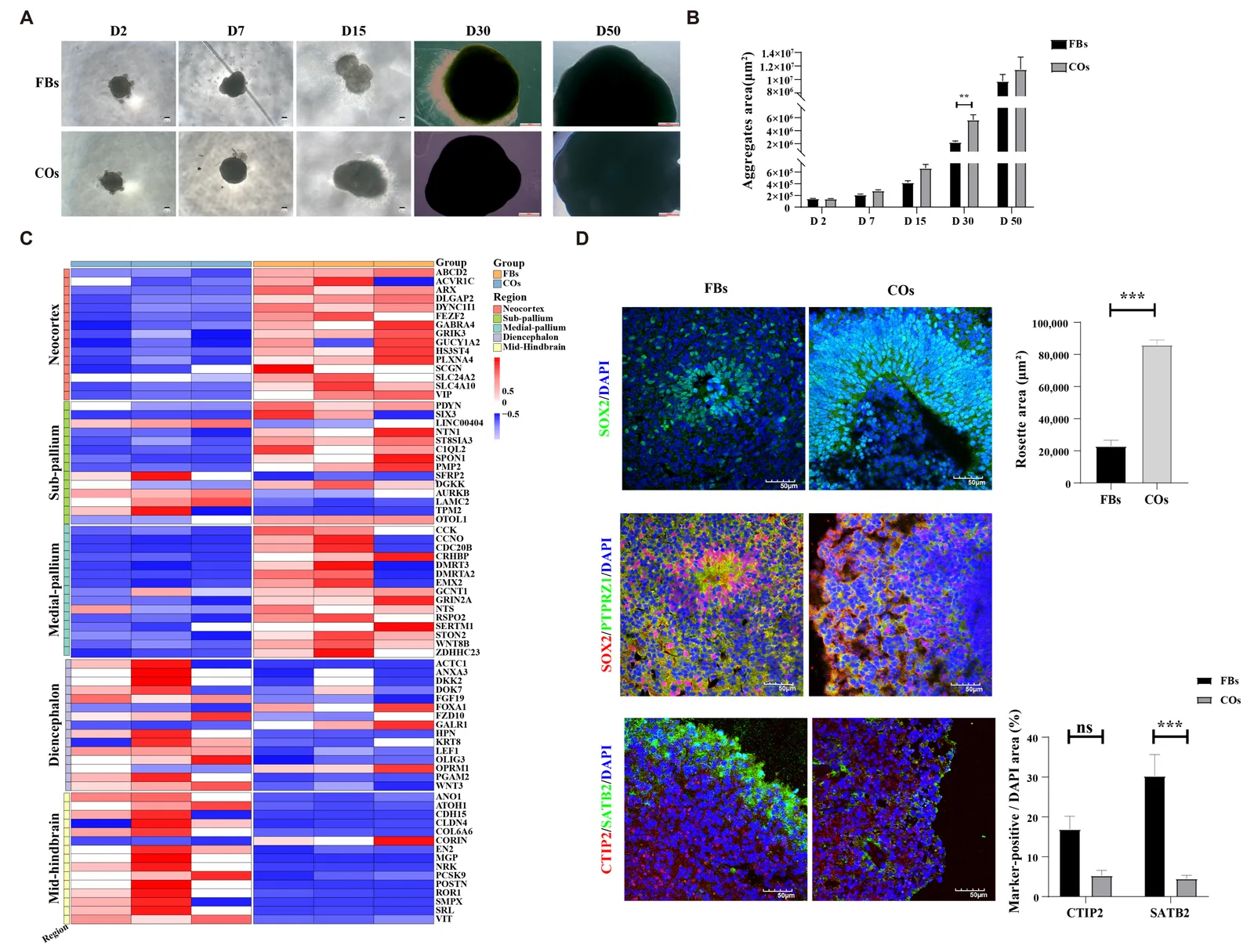
Morphological, molecular, and structural comparisons between human forebrain organoids and cerebral organoids. (**A**) Representative bright-field micrographs showing the morphological development of FBs and COs at indicated time points (Days 2, 7, 15, 30, and 50). Both organoid types formed spherical 3D aggregates and increased in size progressively over the culture period. Scale bars: 100 μm (Days 2, 7, and 15); 500 μm (Day 30 and Day 50). (**B**) Quantitative statistical analysis of organoid aggregate area (μm^2^) between FBs and COs at indicated developmental days. Data are presented as mean ± SEM (*n* = 6–8 per group). Statistical significance was assessed by two-way ANOVA followed by Tukey’s post hoc test. A significant group-by-time interaction was detected (*p* = 0.0164). Post hoc analysis revealed that FBs showed a significantly smaller aggregate area than COs only at Day 30 (adjusted *p*= 0.0044). ** *p* < 0.01. (**C**) Transcriptomic heatmap showing the expression of representative brain region-specific genes, which were identified via differential expression analysis and GSEA of FBs and COs at Day 50 (*n* = 3 independent organoids derived from a single differentiation experiment). The top 15 genes with the highest absolute log_2_ FC values for each brain region were retrieved from GSEA leading-edge signatures. Gene expression was normalized by row-wise Z-score; blue indicates low expression, and red indicates high expression. Genes are grouped by brain region (left annotation), and samples are separated by organoid type (top annotation). FBs showed enriched expression of neocortical and subpallial forebrain genes, whereas COs preferentially expressed midbrain- and hindbrain-associated genes. (**D**) Representative immunofluorescence and quantitative analysis of cellular composition in FBs versus COs at Day 50. Representative immunofluorescence images showed SOX2^+^ neural progenitors, SOX2^+^/PTPRZ1^+^ oRGCs, CTIP2^+^ deep-layer neurons, and SATB2^+^ upper-layer neurons in both organoid groups. FBs exhibited clear cortical lamination, with CTIP2^+^ deep-layer neurons localized internally and SATB2^+^ upper-layer neurons positioned on the outer side. Quantitative analyses of rosette area (populated SOX2^+^ progenitors) were performed using a two-tailed Student’s *t*-test. The area fractions of CTIP2^+^ and SATB2^+^ positive signals were quantified and analyzed by two-way ANOVA followed by Tukey’s post hoc multiple-comparison test. Although FBs showed a trend toward higher CTIP2^+^-positive area relative to COs, this difference did not reach statistical significance (adjusted *p* = 0.0836). SATB2^+^-positive area was significantly increased in FBs (adjusted *p* < 0.001). Quantitative analysis was performed with *n* = 6 independent organoids obtained from a single differentiation experiment. Scale bars: 50 μm. All quantitative data are presented as the mean ± SEM. *** *p* < 0.001 (two-tailed Student’s *t*-test, rosette-area comparison), *** adjusted *p* < 0.001 (Tukey’s post hoc test, SATB2-positive-area comparison), ns, not significant (adjusted *p* > 0.05; CTIP2-positive-area comparison).

To clarify the intrinsic differences between human FBs and COs, we performed parallel culture and conducted multi-dimensional comparative analysis of morphological phenotypes, brain regional identities, cellular composition, and whole-transcriptome profiles, hoping to provide robust experimental evidence for rational organoid selection in neurodevelopmental and pathological studies. Both FBs and COs formed spherical three-dimensional aggregates and exhibited sustained growth throughout the culture period (Figure 4A). Two-way ANOVA detected a significant group-by-time interaction (*p* = 0.0164), indicating divergent growth trajectories between FBs and COs. Quantitative analysis via Tukey’s post hoc test revealed that the aggregate area of FBs was significantly smaller than that of COs at Day 30 (adjusted *p* < 0.01). At the remaining time points (Days 2, 7, 15, and 50), FBs showed moderately smaller aggregate area values relative to COs, yet these differences did not reach statistical significance (Figure 4B). This morphological discrepancy might arise from divergent proliferative behaviors of neural progenitors. Specifically, COs displayed enhanced proliferative activity in neural progenitors that promoted rapid organoid enlargement, whereas the forebrain-specific differentiation program drove progenitor cells to exit the cell cycle and commit to neural fate, thereby attenuating progenitor proliferation in FBs. This divergent proliferative signature was supported by the reduced abundance of neural progenitors and the increased proportion of SATB2^+^ observed in FBs via immunofluorescence (Figure 4D).

To dissect molecular differences in regional fate specification between the two organoid models, bulk RNA sequencing was performed on FBs and COs harvested at Day 50. GSEA and heatmap visualization of brain region-specific gene sets revealed prominent divergence in regional lineage commitment between FBs and COs. For heatmap construction, the top 15 leading-edge genes per brain-region gene set were sorted by absolute log_2_ FC (Figure 4C). FBs showed robust enrichment of canonical forebrain-specific region signatures, including the neocortex, sub-pallium, and medial pallium, indicative of potent activation of core forebrain transcriptional programs in FBs. Conversely, genes upregulated in COs were enriched in midbrain and hindbrain signatures, revealing a predominant posterior lineage preference in COs. These transcriptomic findings confirmed robust forebrain lineage specification in FBs. By contrast, COs displayed broader regional heterogeneity, with relatively stronger enrichment for mid-hindbrain gene signatures compared with FBs.

Immunofluorescence staining was performed to compare cellular composition and tissue architecture between FBs and COs at Day 50 (Figure 4D). Both organoids harbored SOX2^+^ vRGCs that self-organized into VZ-like rosette structures. Notably, COs exhibited significantly larger VZ-like rosette areas compared with FBs (two-tailed Student’s *t*-test, ***, *p* < 0.001). SOX2^+^/PTPRZ1^+^ oRGCs were present in both organoid groups. For neuronal differentiation and cortical lamination, FBs robustly produced CTIP2^+^ deep-layer neurons and SATB2^+^ upper-layer neurons, which organized in a stereotypical inside-out laminar pattern recapitulating the architectural features of the human fetal neocortex. We further quantified the area fractions of deep-layer marker CTIP2 and upper-layer marker SATB2 within organoid sections. A two-way ANOVA was applied followed by Tukey’s post hoc multiple comparison test. Although FBs displayed a trend toward increased CTIP2-positive area fraction relative to COs, this difference did not reach statistical significance (adjusted *p* = 0.0836). In contrast, the SATB2-positive neuron area fraction was significantly elevated in FBs compared with COs (***, adjusted *p* < 0.001). Taken together, immunofluorescence analyses indicated accelerated neuronal differentiation in Day 50 FBs as compared to COs, with diminished neural progenitor abundance, expanded neuronal populations, and enhanced capacity to recapitulate the primary layered structure of human cortical plate.

### 3.4. Enhanced Neuronal Differentiation and Attenuated Proliferation Defined by Divergent Transcriptomic Signatures in Human Forebrain Organoids

**Figure 5:**
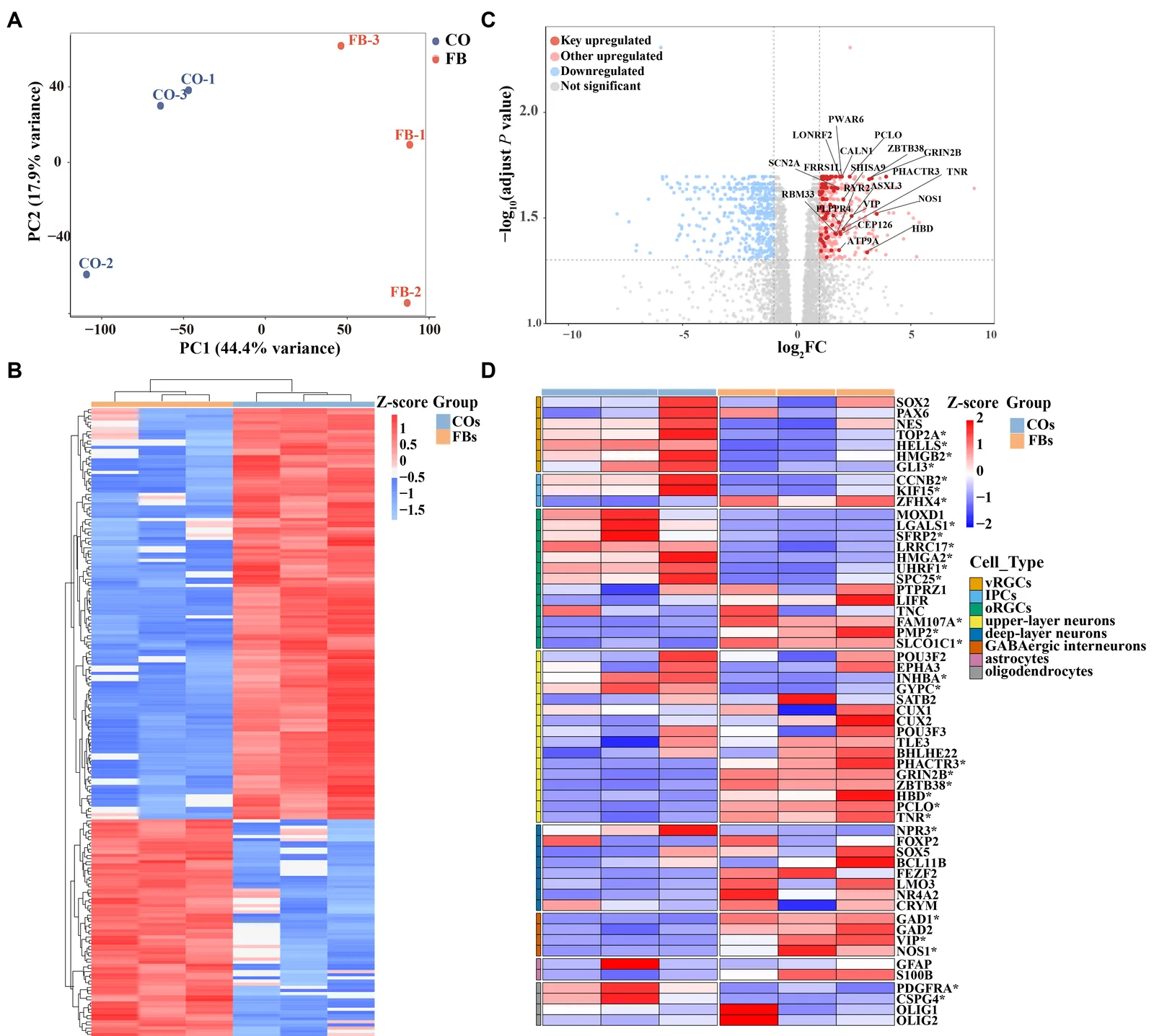
Transcriptomic profiling and differential gene expression analysis of FBs compared with COs at Day 50. (**A**) Principal component analysis (PCA) showing global transcriptional separation between FBs and COs based on voom-normalized transcriptome data. Each dot corresponds to an individual biological replicate. FBs (red, *n* = 3 independent organoids) and COs (blue, *n* = 3 independent organoids) formed independent clusters. The first two principal components individually accounted for 44.4% (PC1) and 17.9% (PC2) of the total transcriptional variance. (**B**) Unsupervised hierarchical clustering heatmap of the top 200 DEGs ranked by |log_2_FC|. Rows represent genes, and columns represent organoid samples. Gene expression values were normalized to row-wise Z-scores and the top annotation bar distinguishes different sample groups. (**C**) Volcano plot illustrating overall DEG distribution between FBs and COs. Genes satisfying the thresholds of |log_2_FC| ≥ 1 and adjusted *p* < 0.05 were regarded as significantly differentially expressed. Red dots indicate upregulated genes, and blue dots indicate downregulated genes, and gray dots represent non-significant genes. Representative signature genes for upper-layer neurons and GABAergic interneurons were labeled. (**D**) Heatmap showing expression pattern of cell type-specific marker genes in FBs and COs. Expression was normalized via Z-score. Asterisks-marked (*) genes indicate significant differential expression (adjusted *p* < 0.05 and |log_2_FC| ≥ 1). The top annotation distinguishes FB and CO groups, whereas the left annotation bar indicates cell-type identity, including vRGCs, IPCs, oRGCs, deep-layer neurons, upper-layer neurons, GABAergic interneurons, astrocytes, and oligodendrocytes. All samples (FBs, *n* = 3; COs, *n* = 3) are independent organoids derived from a single differentiation experiment.

Transcriptomic profiling was performed on Day 50 FBs and COs to dissect molecular differences across the whole-transcriptome level. PCA revealed clear segregation of FBs and COs into two distinct clusters (Figure 5A). The top two principal components (Dim1: 44.4%; Dim2: 17.9%) cumulatively explained 62.3% of total transcriptional variance, demonstrating globally divergent transcriptomic landscapes between the two organoid models. Hierarchical clustering built upon DEG expression profiles further confirmed completely segregated transcriptional landscapes between FBs and COs, with biological replicates tightly clustered within each group (Figure 5B). These findings indicated consistent transcriptomic profiles among independent organoids from a single differentiation batch.

We performed differential expression analysis using the limma–voom pipeline. The mean-variance dispersion relationship indicated appropriate voom weight estimation for our RNA-seq dataset (Supplementary Figure S1). We identified statistically significant DEGs between FBs and COs with a screening threshold of |log_2_FC| ≥ 1 and adjusted *p* < 0.05. A total of 437 upregulated and 492 downregulated genes were identified in FBs compared with COs (Figure 5C). Upregulated DEGs included core forebrain patterning and neuronal maturation regulators (*SHH*, *ARX*), as well as upper-layer neuron and GABAergic interneuron signature genes (*PHACTR3*, *NOS1*, *GRIN2B*, *ZBTB38*, *HBD,* and *VIP*) [30,31]. Conversely, genes downregulated in FBs primarily comprised canonical modulators associated with posterior neural specification (*HOXA9*, *HOXB5*) and cell proliferation (*TBX3* and *HMGA2*).

Furthermore, we systematically profiled cell-type-specific transcriptomic signatures to explore transcriptional differences in cell-type-associated gene sets between the two organoid models. As shown in Figure 5D, COs displayed elevated transcript levels of signature genes marking vRGCs, IPCs, and partial oRGC subsets. In comparison, FBs exhibited high expression of canonical marker gene sets for upper-layer neurons. Overall, these transcriptomic signatures indicated robust forebrain-associated transcriptional programs in FBs, accompanied by transcriptional features consistent with reduced progenitor proliferation and advanced neuronal differentiation. These results reveal a pronounced transcriptional shift toward neuronal differentiation at the transcriptomic level in FBs compared with COs. These transcript-level findings are largely consistent with preceding quantitative immunofluorescence observations of reduced rosette area (SOX2^+^ progenitors) and significantly elevated SATB2-positive upper-layer neurons in FBs (Figure 4D).

**Figure 6:**
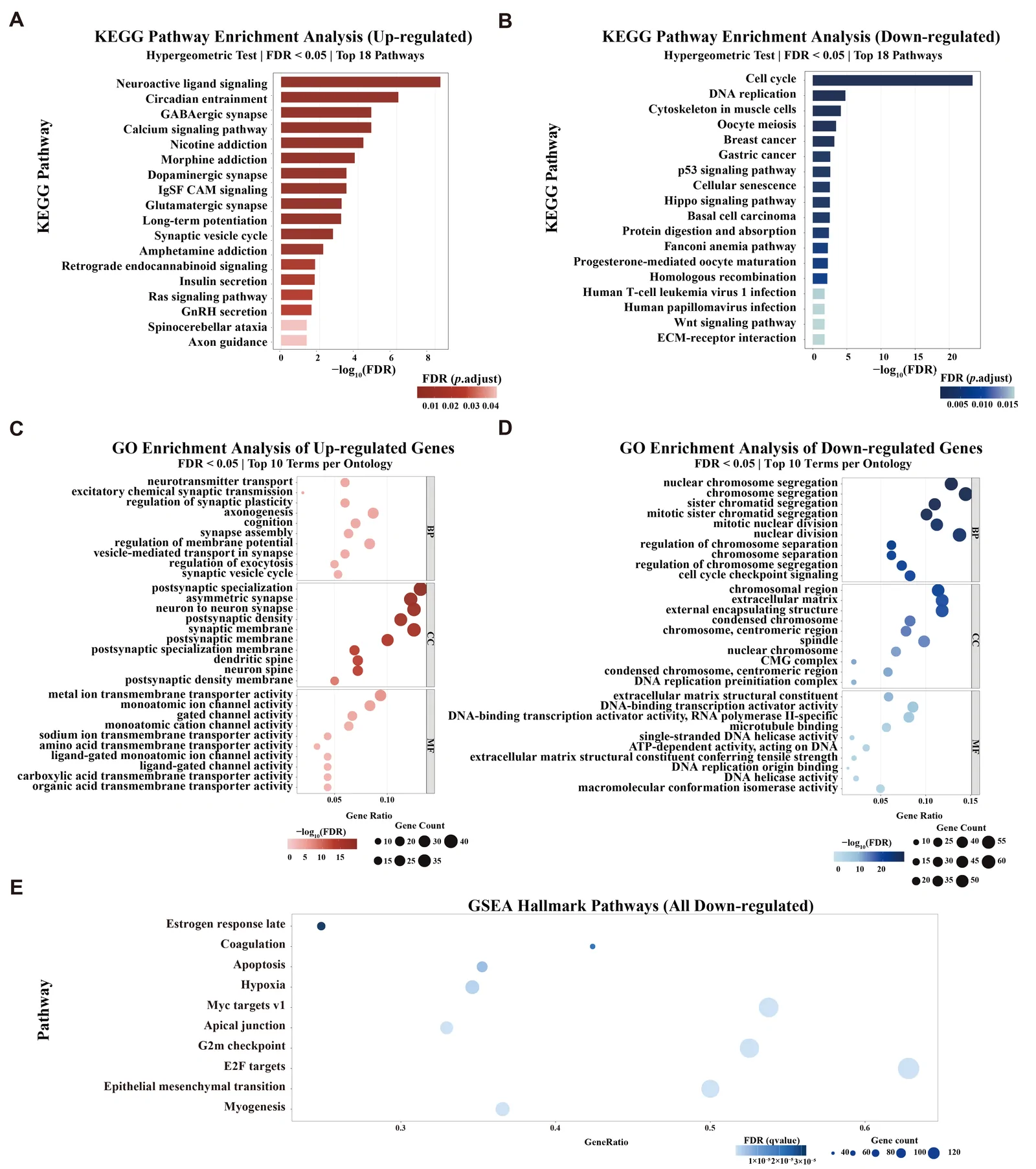
Functional enrichment analysis of differentially expressed genes between FBs and COs at Day 50. (**A**,**B**) KEGG pathway enrichment analysis of up-regulated (**A**) and down-regulated (**B**) genes. Color intensity corresponds to −log_10_ transformed adjusted *p* (FDR). Pathways with FDR < 0.05 are shown. (**C**,**D**) GO term enrichment analysis for up-regulated (**C**) and down-regulated (**D**) DEGs. Bubble plots illustrate the top 10 most significantly enriched terms across three core GO categories: biological process (BP), cellular component (CC), and molecular function (MF). The *x*-axis represents the gene ratio, bubble size indicates the number of genes, and color gradient represents the adjusted *p* (FDR). (**E**) GSEA hallmark pathway enrichment analysis. The bubble plot shows all significantly enriched hallmark pathways, which were all down-regulated. The *x*-axis represents the gene ratio, bubble size corresponds to the number of core enriched genes, and color intensity indicates the FDR. All enrichment analyses were performed with FDR correction, and FDR < 0.05 was considered statistically significant.

We next performed KEGG and GO enrichment analyses on upregulated and downregulated DEGs to characterize developmental and functional differences between FBs and COs, followed by GSEA using the full transcriptomic dataset (Figure 6A–E). KEGG pathway enrichment of upregulated DEGs in FBs identified significant enrichment in neuronal signaling cascades, primarily encompassing neuroactive ligand signaling, GABAergic synapses, dopaminergic synapses, glutamatergic synapses, LTP, and the calcium signaling pathway (Figure 6A). LTP represents a well-characterized form of synaptic plasticity underlying the molecular machinery of learning and memory [32,33]. These transcriptional signatures indicated that synaptic-associated transcriptional programs were enhanced in FBs as compared to COs. Of note, several KEGG terms annotated as “addiction” emerged in our top enriched pathways. These gene sets share substantial overlap with common neurotransmitter-receptor and calcium-signaling gene modules, meaning this enrichment does not indicate addiction-specific biological programs in FBs. Rather, it likely mirrors elevated neuronal differentiation and synaptic activity, supporting the potential utility of this FB model for evaluating addictive compounds. GO enrichment analysis across three core ontology categories further delineated the biological characteristics of FBs (Figure 6C). Upregulated DEGs were predominantly enriched in synaptic-related terms, covering neurotransmitter transport, excitatory synaptic transmission, and synaptic plasticity regulation. BP terms were enriched in neurotransmitter transport, synaptic transmission, and regulation of synaptic plasticity; CC terms prioritized postsynaptic specialization, asymmetric synapses, and neuron to neuron synapses; MF terms revolved around ion channel and transmembrane transporter activities, critical for maintaining neuronal excitability. Combined KEGG and GO enrichment results collectively revealed that transcriptional programs linked to synaptic signaling, synaptic structure, and neurotransmitter-associated gene sets were upregulated in Day 50 FBs relative to COs. On the other hand, FB-downregulated DEGs were enriched for cell cycle progression, DNA replication, and mitotic division pathways (Figure 6B,D). Hallmark GSEA further identified significant suppression of proliferation-related gene sets in FBs, including E2F targets, G2m checkpoint, and Myc targets v1 (Figure 6E), corroborating attenuated cell cycle activity in FBs. Additional suppressed hallmark terms included epithelial-mesenchymal transition and myogenesis, indicating restrained non-neural transcriptional programs. Coordinated downregulation of proliferative and non-neural circuits directs committed forebrain neuronal differentiation in FBs.

In summary, Day 50 FBs exhibited a dual transcriptional regulatory pattern relative to COs, characterized by robust up-regulation of synaptic- and neuronal-associated gene-set pathways and repression of cell cycle and proliferative programs. This unique transcriptomic signature reflected that FBs underwent a pronounced transcriptional shift toward neuronal differentiation and synaptic-signaling-related molecular programs, highlighting FBs as a reliable model for exploring the intrinsic molecular mechanisms underlying synaptic pathology.

### 3.5. Enhanced Synaptic Plasticity Transcriptional Landscapes in Human Forebrain Organoids

**Figure 7:**
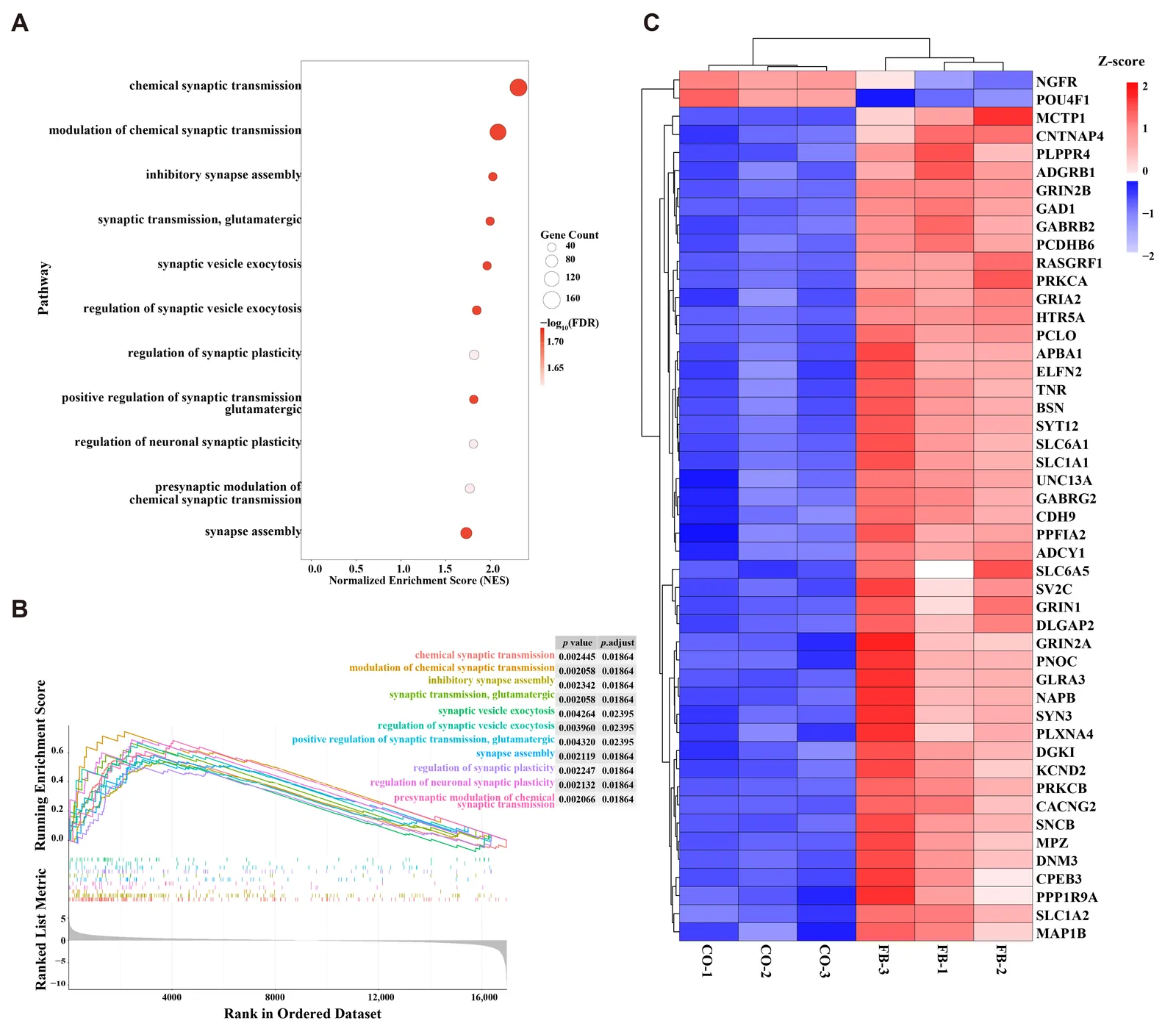
Synaptic plasticity and neural circuit-related transcriptional differences between FBs and COs at Day 50. (**A**) Bubble plot of all significantly enriched synaptic plasticity and neural circuit gene sets identified by pre-ranked GSEA. The *x*-axis represents normalized NES; pathways with NES > 0 were preferentially enriched in FBs. The *y*-axis lists enriched GO/SynGO functional pathways ordered by NES magnitude. Bubble size corresponds to the total number of annotated genes within each pathway (range: 40–160), and bubble color gradient encodes enrichment significance as −log_10_ FDR. All displayed synaptic functional pathways were significantly enriched in FBs. (**B**) Running enrichment score curves for all FDR-significant synaptic and neural circuit-related gene sets. Each colored curve represents an individual pathway. Vertical tick bars below the curves mark the rank positions of core enriched leading-edge genes in the full pre-ranked transcriptome. Adjusted *p*-values (*p*.adjust) of each pathway are annotated. (**C**) Heatmap of synaptic-related DEGs extracted from GSEA-significantly enriched synaptic pathways between FBs and COs at Day 50. Rows represent individual genes, and columns correspond to biological replicates of the COs and FBs. The color gradient from blue to red indicates relative low-to-high normalized gene expression levels. All plotted genes satisfied differential expression thresholds: FDR < 0.05 and |log_2_FC| ≥ 1. Hierarchical clustering revealed distinct transcriptional patterns between the two organoid groups.

We performed targeted GSEA to interrogate synaptic and neural-circuit-related transcriptional signatures in FBs. Core pathways governing chemical synaptic transmission, synapse assembly, regulation of synaptic plasticity, synaptic vesicle exocytosis, and synaptic plasticity were significantly enriched in FBs (Figure 7A,B). Notably, heatmap analysis of core synaptic DEGs revealed consistent upregulation of genes encoding key components involved in excitatory and inhibitory synaptic transmission, including glutamatergic excitatory synapses subunits (*GRIN1*, *GRIN2A*, *GRIN2B*), GABAergic inhibitory synapses-related molecules (*GAD1*, *GABRB2*, *GABRG2*), and synaptic vesicle trafficking and exocytosis factors (*PRKCB*, *PCLO*, *SYN3*, *SYT12*, *SV2C*) (Figure 7C). We also identified multiple modulators for synapse assembly and synaptic transmission, such as *PRKCA*, *PCDHB6*, *CNTNAP4*, *PLPPR4*, *HTR5A*, *APBA1*, *ELFN2*, and *BSN*. Furthermore, *CAMK2A*, the core regulator of LTP, also showed significant upregulation in FBs, together with a panel of genes implicated in neuronal synaptic plasticity (*MCTP1*, *ADGRB1*, *RASGRF1*) [34,35]. At the molecular level, these genes collectively orchestrate synapse assembly, balanced excitatory-inhibitory neurotransmission, and activity-dependent long-term synaptic remodeling. In contrast, a small subset of genes such as *NGFR* and *POU4F1* displayed preferential upregulation in COs, which mainly participate in presynaptic modulation of chemical synaptic transmission and synapse assembly.

Collectively, coordinated transcriptional activation of gene sets associated with synaptic transmission and synaptic plasticity at both the pathway and individual gene levels demonstrated that FBs harbored comprehensive molecular prerequisites for robust synaptogenesis and synaptic plasticity. These findings support the suggestion that FBs provide a valuable in vitro organoid model for dissecting the molecular mechanisms underlying human forebrain development and synaptic dysfunction-associated neurodevelopmental disorders.

## 4. Discussion

Human cerebral organoids spontaneously generated stochastic anterior-posterior patterning, mixed regional identities, and disorganized cortical plate stratification, restricting their application for studying human forebrain synaptogenesis and related neurodevelopmental disorders. In the present study, we generated a robust FB system using triple inhibition patterning cues, with modifications to the protocol for COs. Comprehensive morphological, immunofluorescent, and transcriptomic characterizations collectively demonstrated that FBs exhibited authentic forebrain identity, a developmental shift from proliferative progenitor expansion toward neuronal differentiation, and the presence of oRGCs reminiscent of the developing human fetal neocortex [36]. Transcriptomic comparison between FBs and COs further established preliminary evaluative benchmarks to guide rational organoid selection for targeted disease modeling and mechanistic research.

The triple-inhibitor strategy effectively suppressed ectopic posterior and non-neural lineage differentiation, enabling stringent forebrain fate commitment. FBs consistently exhibited canonical forebrain markers (FOXG1, EMX2 and PAX6) and elevated forebrain (neocortex, sub-pallium and medial-pallium) signature genes, whereas posterior patterning regulators (*HOXA9*, *HOXB5*) and non-neural transcriptional programs associated with mesendodermal differentiation, epithelial-mesenchymal transition, and myogenesis were markedly repressed. This robust restriction of off-target lineages endowed FBs with high regional homogeneity, providing a reliable platform for investigating human cortical progenitor diversification and ordered neocortical development. Compared with previously reported single- or dual-inhibitor protocols, our system achieved more specific forebrain specification and minimized posterior contamination. While retaining the effective triple-inhibition strategy, our protocol flexibly adopts a simplified basal culture workflow largely consistent with classical unguided cerebral organoid protocols [12,22]. This design supports robust forebrain identity and simplifies experimental manipulations, with the potential to reduce organoid heterogeneity.

We further established a multi-layered validation framework integrating morphological, immunofluorescent, and transcriptomic analyses, comprehensively characterizing FB-specific developmental features beyond morphology alone. Importantly, we identified significant transcriptional activation of synaptic developmental programs in Day 50 FBs, expanding their applicability for studying neural differentiation and synaptopathy. At Day 50, FBs displayed an earlier developmental transition from proliferative expansion to neuronal differentiation compared with COs. COs retained enlarged VZ-like rosettes with robust vRGCs proliferation, whereas FBs showed diminished rosette areas and markedly expanded SATB2-positive upper-layer neuron populations, indicating accelerated neuronal differentiation. Consistent with prior reports describing abundant oRGC enrichment in FBs [22], our FBs also harbored oRGC populations. These oRGCs are critical for neocortical expansion in the developing human fetal forebrain [37]. Notably, our work extends these prior observations by directly benchmarking FBs against classical COs at the identical culture time point, revealing both accelerated neuronal differentiation and enhanced synapse-associated transcriptional programs. In addition, FBs developed ordered layered structures recapitulating the canonical inside-out patterning of the human neocortex, with deep-layer CTIP2^+^ neurons positioned internally and SATB2^+^ upper-layer neurons distributed peripherally. KEGG, GO, and hallmark GSEA analyses further delineated the transcriptional pathways driving the proliferation-to-differentiation switch in FBs. Genes upregulated in FBs were predominantly enriched in synaptic transmission, calcium signaling, and synaptic plasticity-related pathways, whereas cell cycle, DNA replication, and proliferation gene sets (E2F, G2/M checkpoint, Myc targets) were significantly suppressed. Collectively, progenitors within FBs undergo precocious cell-cycle exit, exiting sustained proliferative expansion earlier to initiate neuronal differentiation. Consistently, core synaptic genes associated with excitatory/inhibitory synapse assembly, vesicle trafficking, and postsynaptic scaffolding were coordinately elevated in FBs, providing transcriptional evidence for early neural differentiation and synaptogenesis.

Despite the strengths of our FB model in achieving homogeneous forebrain specification and precocious acquisition of synaptic transcriptional signatures, several inherent limitations warrant further consideration. First, this article demonstrated that FBs exhibit relatively early neuronal differentiation and enhanced synapse-associated transcriptional programs, based solely on immunofluorescence and transcriptomic profiling. Follow-up functional assays including electrophysiological recording, transmission electron microscopy, and calcium imaging assays will be required to confirm whether the upregulated synaptic developmental transcripts in FBs correspond to electrophysiologically competent synapses and physiologically functional neural circuits. Second, transcriptomic profiling comparing FBs and COs was performed at a single developmental time point (Day 50), using independent organoids derived from a single differentiation experiment. These biological replicates are individual organoids within this differentiation run, rather than organoids generated from separate, independent differentiation batches. Longitudinal transcriptomic tracking spanning successive differentiation stages would help delineate the temporal cascade of forebrain fate specification and further disentangle transcriptional signatures driven by regional identity versus neuronal maturation in future investigations. In addition, bulk RNA-seq based marker-gene analysis cannot formally distinguish whether altered marker expression arises from shifts in cell-type proportions or from cell-intrinsic transcriptional changes within individual cell populations. Bulk transcriptomic signatures can only serve as preliminary supportive evidence for inferring potential cellular-composition differences, rather than as definitive proof of altered cell-type abundance. Future work incorporating single-cell RNA-sequencing together with validated computational deconvolution approaches will be required to rigorously resolve cell-type compositional differences between FBs and COs. Third, brain organoids inherently lack vascularization, resident immune populations, and the intricate three-dimensional spatial microenvironment of the native human fetal brain, inevitably creating a biological gap between in vitro organoid systems and bona fide in vivo neurodevelopment. To mitigate these limitations, future strategies may include co-culturing organoids with endothelial cells or xenotransplantation into rodents to generate vascularized constructs [38], introducing microglial progenitors to recapitulate neuroimmune crosstalk, and utilizing microfluidic organ-on-a-chip devices to spatiotemporally manipulate signaling gradients for precise regional patterning and elaborate neural circuit assembly [38,39,40]. Furthermore, the native forebrain relies on reciprocal anatomical and functional connectivity with multiple adjacent brain regions to support complete cortical maturation and neural circuit assembly, interactions that cannot be recapitulated in isolated single-region FBs [41,42,43,44]. Accordingly, a growing number of studies have therefore prioritized the production of region-specified brain organoids and the fabrication of fused multi-region assembloids, in which preserved regional authenticity permits systematic exploration of inter-brain-region neural crosstalk [45,46,47].

Our FB model provides a homogeneous forebrain system with prominent upregulation of synaptic-related transcriptional programs, enabling precise dissection of core mechanisms underlying human forebrain patterning and synaptogenesis processes inaccessible to direct human embryonic tissues. For translational research, FBs hold prominent potential for constructing patient-derived disease models of forebrain-related neurodevelopmental disorders, including autism spectrum disorder, schizophrenia, and intellectual disability, supporting pathogenic mechanism exploration and candidate therapeutic screening. Furthermore, the transcriptome-based evaluative criteria proposed herein provide a practical guideline for the rational selection of region-specified organoids, facilitating the standardization of organoid-oriented neuroscience research. Future work will focus on generating isogenic disease models to unravel synaptic pathological alterations. Collectively, our FBs represent a reliable in vitro paradigm that enables sophisticated mechanistic investigations of human forebrain ontogeny and the etiology of synapse-associated neurological disorders.

## 5. Conclusions

In this study, we generated human forebrain organoids using a triple-inhibition (dual SMAD/Wnt) strategy. These FBs stably maintained forebrain regional identity, diverse forebrain cell populations, and rudimentary cortical laminar architecture during long-term culture. In contrast to classical COs, which exhibited substantial regional heterogeneity and prominent mid-hindbrain lineage bias, our forebrain organoids acquired robust forebrain regional specificity. Furthermore, FBs exhibited enhanced neuronal differentiation and elevated expression of synapse-related transcripts, accompanied by up-regulated synaptic-associated pathways, when compared to COs at Day 50. At this time point, classical COs retained higher progenitor signatures with sustained proliferative transcriptional activity. Direct side-by-side transcriptomic and phenotypic comparisons between the two organoid systems provide practical guidance for experimental model selection. FBs are well-suited to use in investigating forebrain neuron biology and synaptopathy-associated disease mechanisms. Classical COs are appropriate for studies that demand broader brain regional diversity and prominent proliferative properties, such as transplantation-based translational research. Collectively, this well-characterized forebrain organoid platform enables reliable organoid generation and serves as a valuable in vitro tool for dissecting molecular mechanisms underlying human forebrain synaptic disorders.

## Data Availability

Raw RNA-sequencing data reported in this study have been deposited in the Gene Expression Omnibus (GEO) database under accession number GSE346531. The dataset will be made publicly available upon online publication of this article.

## Supplementary Materials

The following supporting information can be downloaded at: https://www.mdpi.com/article/10.3390/cells15201842/s1, Table S1: Comparison of organoid induction protocols; Table S2: Antibody information used for immunofluorescence staining; Table S3: Sample metadata for RNA-seq datasets; Table S4: Canonical forebrain cell-type-specific gene signatures; Table S5: Human fetal brain region-specific gene sets (12–24 pcw) derived from published databases and Allen Human Brain Atlas; Table S6: Synaptic gene set for functional enrichment analysis integrated from Gene Ontology entries and SynGO database; Figure S1 voom mean variance plot.

## Abbreviations

The following abbreviations are used in this manuscript:

COs	Cerebral Organoids
FBs	Forebrain Organoids
TGF-β	Transforming Growth Factor-β
vRGCs	Ventricular Radial Glial Cells
oRGCs	Outer Radial Glial Cells
IPCs	Intermediate Progenitor Cells
VZ	Ventricular Zone
SVZ	Subventricular Zone
LTP	Long-term Potentiation

## References

[1] Yang H., Wu G., Li Y., Xu X., Cong J., Xu H., Ma Y., Li Y., Chen R., Pines A. (2025). Connectional axis of individual functional variability: Patterns, structural correlates, and relevance for development and cognition. Proc. Natl. Acad. Sci. USA.

[2] Hirai S., Miwa H., Shimbo H., Nakajima K., Kondo M., Tanaka T., Ohtaka-Maruyama C., Hirai S., Okado H. (2023). The mouse model of intellectual disability by haploinsufficiency shows cognitive dysfunction with synaptic impairment. Mol. Psychiatr..

[3] Wang L., Wang B., Wu C., Wang J., Sun M. (2023). Autism Spectrum Disorder: Neurodevelopmental Risk Factors, Biological Mechanism, and Precision Therapy. Int. J. Mol. Sci..

[4] Jiang C.-C., Lin L.-S., Long S., Ke X.-Y., Fukunaga K., Lu Y.-M., Han F. (2022). Signalling pathways in autism spectrum disorder: Mechanisms and therapeutic implications. Signal Transduct. Target. Ther..

[5] Velasco S., Kedaigle A.J., Simmons S.K., Nash A., Rocha M., Quadrato G., Paulsen B., Nguyen L., Adiconis X., Regev A. (2019). Individual brain organoids reproducibly form cell diversity of the human cerebral cortex. Nature.

[6] Kelley K.W., Pașca S.P. (2022). Human brain organogenesis: Toward a cellular understanding of development and disease. Cell.

[7] Lancaster M.A., Knoblich J.A. (2014). Organogenesis in a dish: Modeling development and disease using organoid technologies. Science.

[8] Wang L., Nakamura Y., Li J., Sievert D., Liu Y., Nguyen T., Jetti P.S., Thai E., Zhou R.Y., Weng J. (2025). A phenotypic brain organoid atlas and biobank for neurodevelopmental disorders. Cell Stem Cell.

[9] Yuan N.Y., Richards W.D., Parham K.T., Clark S.G., Lebakken C.S. (2026). Stimulus-Based ApoE Alzheimer’s Disease Induction Model Using Microglia-Containing Brain Organoids for Drug Discovery. Cells.

[10] Han J.J. (2023). FDA Modernization Act 2.0 allows for alternatives to animal testing. Artif. Organs.

[11] Mullard A. (2025). Is the FDA’s plan to phase out animal toxicity testing realistic?. Nat. Rev. Drug Discov..

[12] Lancaster M.A., Renner M., Martin C.-A., Wenzel D., Bicknell L.S., Hurles M.E., Homfray T., Penninger J.M., Jackson A.P., Knoblich J.A. (2013). Cerebral organoids model human brain development and microcephaly. Nature.

[13] Kelava I., Lancaster M.A. (2016). Stem Cell Models of Human Brain Development. Cell Stem Cell.

[14] Tang X.-Y., Wu S., Wang D., Chu C., Hong Y., Tao M., Hu H., Xu M., Guo X., Liu Y. (2022). Human organoids in basic research and clinical applications. Signal Transduct. Target. Ther..

[15] Ladewig J., Koch P., Iefremova V., Jabali A., Krefft O. (2018). Generation of Standardized and Reproducible Forebrain-type Cerebral Organoids from Human Induced Pluripotent Stem Cells. J. Vis. Exp..

[16] Sloan S.A., Andersen J., Pașca A.M., Birey F., Pașca S.P. (2018). Generation and assembly of human brain region–specific three-dimensional cultures. Nat. Protoc..

[17] Yoon S.-J., Elahi L.S., Pașca A.M., Marton R.M., Gordon A., Revah O., Miura Y., Walczak E.M., Holdgate G.M., Fan H.C. (2018). Reliability of human cortical organoid generation. Nat. Methods.

[18] Ullah H.M.A., Huang Q.J., Chiola S., Wang Y.Q., Shcheglovitov A. (2026). Generating and characterizing human telencephalic brain organoids from stem cell-derived single neural rosettes. Nat. Protoc..

[19] Wang S., Wang Z., Wang X., Zhang X., Xu T., Miao C. (2023). Humanized cerebral organoids-based ischemic stroke model for discovering of potential anti-stroke agents. Acta Pharmacol. Sin..

[20] Li W., Sun W., Zhang Y., Wei W., Ambasudhan R., Xia P., Talantova M., Lin T., Kim J., Wang X. (2011). Rapid induction and long-term self-renewal of primitive neural precursors from human embryonic stem cells by small molecule inhibitors. Proc. Natl. Acad. Sci. USA.

[21] Chen G.K., Hou Z.G., Gulbranson D.R., Thomson J.A. (2010). Actin-Myosin Contractility Is Responsible for the Reduced Viability of Dissociated Human Embryonic Stem Cells. Cell Stem Cell.

[22] Rosebrock D., Arora S., Mutukula N., Volkman R., Gralinska E., Balaskas A., Aragonés Hernández A., Buschow R., Brändl B., Müller F.-J. (2022). Enhanced cortical neural stem cell identity through short SMAD and WNT inhibition in human cerebral organoids facilitates emergence of outer radial glial cells. Nat. Cell Biol..

[23] Xiang Y., Cakir B., Park I.-H. (2021). Deconstructing and reconstructing the human brain with regionally specified brain organoids. Semin. Cell Dev. Biol..

[24] Chambers S.M., Fasano C.A., Papapetrou E.P., Tomishima M., Sadelain M., Studer L. (2009). Highly efficient neural conversion of human ES and iPS cells by dual inhibition of SMAD signaling. Nat. Biotechnol..

[25] Lancaster M.A., Knoblich J.A. (2014). Generation of cerebral organoids from human pluripotent stem cells. Nat. Protoc..

[26] Kang H.J., Kawasawa Y.I., Cheng F., Zhu Y., Xu X., Li M., Sousa A.M.M., Pletikos M., Meyer K.A., Sedmak G. (2011). Spatio-temporal transcriptome of the human brain. Nature.

[27] Koopmans F., van Nierop P., Andres-Alonso M., Bymes A., Cijsouw T., Coba M.P., Cornelisse L.N., Farrell R.J., Goldschmidt H.L., Howrigan D.P. (2019). SynGO: An Evidence-Based, Expert-Curated Knowledge Base for the Synapse. Neuron.

[28] Zhang X.-P., Wang X.-Y., Wang S.-N., Miao C.-Y. (2023). The generation and properties of human cortical organoids as a disease model for malformations of cortical development. Neural Regen. Res..

[29] Yi S.M., Huang M., Xian C.M., Kong X., Yin S.G., Peng J.H., Li H.D., Jiang Y., Xie B.Q., Xie H.F. (2026). Hedgehog signaling activation rescues hypoxia-induced ferroptosis in human brain organoids. Neural Regen. Res..

[30] Ding J.W., Kim C.N., Ostrowski M.S., Abeykoon Y., Pavlovic B.J., Wallace J.L., Schaefer N.K., Nowakowski T.J., Pollen A.A. (2026). Dissecting gene regulatory networks governing human cortical cell fate. Nature.

[31] Lim Y., Cho I.-T., Shi X., Grinspan J.B., Cho G., Golden J.A. (2019). Arx Expression Suppresses Ventralization of the Developing Dorsal Forebrain. Sci. Rep..

[32] Bliss T.V.P., Collingridge G.L., Kaang B.-K., Zhuo M. (2016). Synaptic plasticity in the anterior cingulate cortex in acute and chronic pain. Nat. Rev. Neurosci..

[33] Park P., Volianskis A., Sanderson T.M., Bortolotto Z.A., Jane D.E., Zhuo M., Kaang B.-K., Collingridge G.L. (2014). NMDA receptor-dependent long-term potentiation comprises a family of temporally overlapping forms of synaptic plasticity that are induced by different patterns of stimulation. Philos. Trans. R. Soc. B Biol. Sci..

[34] Luo R.C. (2026). Chaperone-mediated autophagy as a sex-specific modulator of synaptic proteostasis and neural function. Autophagy.

[35] Rellos P., Pike A.C.W., Niesen F.H., Salah E., Lee W.H., Von Delft F., Knapp S. (2010). Structure of the CaMKIIδ/Calmodulin Complex Reveals the Molecular Mechanism of CaMKII Kinase Activation. PLoS Biol..

[36] Zhou Y., Song H., Ming G.-L. (2023). Genetics of human brain development. Nat. Rev. Genet..

[37] Pollen A.A., Nowakowski, Tomasz J., Chen J., Retallack H., Sandoval-Espinosa C., Nicholas, Cory R., Shuga J., Liu S.J. (2015). Molecular Identity of Human Outer Radial Glia during Cortical Development. Cell.

[38] Revah O., Gore F., Kelley K.W., Andersen J., Sakai N., Chen X., Li M.-Y., Birey F., Yang X., Saw N.L. (2022). Maturation and circuit integration of transplanted human cortical organoids. Nature.

[39] Pașca S.P., Arlotta P., Bateup H.S., Camp J.G., Cappello S., Gage F.H., Knoblich J.A., Kriegstein A.R., Lancaster M.A., Ming G.-L. (2024). A framework for neural organoids, assembloids and transplantation studies. Nature.

[40] Wang M., Zhang L., Novak S.W., Yu J., Gallina I.S., Xu L.L., Lim C.K., Fernandes S., Shokhirev M.N., Williams A.E. (2024). Morphological diversification and functional maturation of human astrocytes in glia-enriched cortical organoid transplanted in mouse brain. Nat. Biotechnol..

[41] Ananth M.R., Rajebhosale P., Kim R., Talmage D.A., Role L.W. (2023). Basal forebrain cholinergic signalling: Development, connectivity and roles in cognition. Nat. Rev. Neurosci..

[42] Allen S.J., Morishita H. (2024). Local and long-range input balance: A framework for investigating frontal cognitive circuit maturation in health and disease. Sci. Adv..

[43] Leung B.K., Chieng B., Becchi S., Balleine B.W. (2024). A ventral pallidal-thalamocortical circuit mediates the cognitive control of instrumental action. Curr. Biol..

[44] Wagner M.J., Luo L.Q. (2020). Neocortex-Cerebellum Circuits for Cognitive Processing. Trends Neurosci..

[45] Kshirsagar A., Mnatsakanyan H., Kulkarni S., Guo J., Cheng K., Ofria L.D., Bohra O., Sagar R., Mahairaki V., Badr C.E. (2025). Multi-Region Brain Organoids Integrating Cerebral, Mid-Hindbrain, and Endothelial Systems. Adv. Sci..

[46] Andersen J., Revah O., Miura Y., Thom N., Amin N.D., Kelley K.W., Singh M., Chen X., Thete M.V., Walczak E.M. (2020). Generation of Functional Human 3D Cortico-Motor Assembloids. Cell.

[47] Fernando M., Lee S., Wark J.R., Xiao D., Lim B.Y., O’Hara-Wright M., Kim H.J., Smith G.C., Wong T., Teber E.T. (2022). Differentiation of brain and retinal organoids from confluent cultures of pluripotent stem cells connected by nerve-like axonal projections of optic origin. Stem Cell Rep..

